# Dietary intake with supplementation of vitamin D, vitamin B6, and magnesium on depressive symptoms: a public health perspective

**DOI:** 10.3389/fpubh.2024.1369666

**Published:** 2024-03-27

**Authors:** Rohitha Rajasekar, Julia VanderMolen, Katie Barnhart, Nicole Anguilim

**Affiliations:** Grand Valley State University, Grand Rapids, MI, United States

**Keywords:** depression, adults, prevention, vitamins, supplements

## Abstract

**Objective:**

This study aims to understand the impact of dietary intake through supplementation of vitamins D, B6, and magnesium on elevated depressive symptoms, a mental health illness that is a leading contributor to global disability and a public health concern.

**Methods:**

Multiple datasets from the National Health and Nutrition Examination Survey 2017-March 2020 investigated the associations between vitamin D, B6, and magnesium on depression screening scores. A cross-sectional sample of adults over 20 was extracted (*n* = 9,232). Chi-square tests and logistic regression analyses were used to investigate the associations.

**Results:**

Individuals with low amounts of vitamin D (*p* = 0.0481) were more likely to report elevated depressive symptoms relative to those with low amounts of vitamin B6 (*p* = 0.0225). These results remained significant among those with high magnesium (*p* = 0.0133) proportionate to high vitamin B6 (*p* = 0.0225). In the age-adjusted model, a lower intake of vitamin D, vitamin B6, and magnesium showed a relationship with elevated depressive symptoms (Vitamin D: OR = 0.611, 95% CI 0.382–0.980 Vitamin B6: OR = 0.503, 95% CI 0.291–0.867 Magnesium: OR = 0.458, 95% CI 0.277–0.759). The fully adjusted regression model (gender, race/ethnicity, and household food security) showed that a lower intake of vitamin B6 and magnesium correlated with elevated depressive symptoms (Vitamin B6: OR = 0.439, 95% CI 0.260–0.738 Magnesium: OR = 0.465, 95% CI 0.303–0.714).

**Conclusion:**

Preventive measures could be addressed by identifying the risks of vitamin deficiencies. Further epidemiological research is needed for the individual effects of vitamin supplementation and depression screening scores. Future prospective cohort studies exploring these associations, focusing on daily dietary intake, are needed to validate the direction of causation further and understand the underlying mechanisms.

## 1 Introduction

Nutrition is defined as “consuming, absorbing, and using nutrients from the food necessary for growth, development, and maintenance of life” (1). Additionally, nutritional psychiatry is defined as a “specialized field of lifestyle medicine that focuses on the relationship between nutrition and mental health symptoms” (2, 3). Nutritional psychiatry uses food and supplements as alternative mental health disorder treatments (2, 3). It is widely known that eating nutritionally dense food plays a key role in one’s physical health (2–4). The prevalence of harmful mental health outcomes in adults is increasing even with reports of the benefits of micronutrients and food on mental health (4, 5).

As of 2023, depression affects approximately 280 million people worldwide and together with other mental illnesses comprises a leading contributor to global disability (6, 7). Research provides evidence of the relationship between diet and well-being as well as the association specific to effects associated with the interaction of diet and mental health (8). Promoting healthy eating habits and identifying individual dietary components can improve mental health (8–15). Current medications available for the treatment of common psychiatric disorders include vitamins and minerals, which are effective compared to traditional antidepressants in many studies (8). The opposite is seen in studies where an unbalanced diet increases the risk of disease and cognitive decline (16). Additionally, inadequate nutrient intake, poor dietary quality, and obesity are closely related to mood disorders and stress regulation (2, 8, 16).

A growing literature has focused on potential risks associated with mental disorders (2, 9, 16–19). More non-communicable chronic diseases are related to modern lifestyle habits, such as poor dietary patterns containing refined sugars, trans-fatty acids, and limited consumption of plant-based foods (17). Due to these reasons, the interest in nutritional psychiatry is growing and moving to understand the relationship between dietary factors and depression (17, 20). Overall dietary pattern recommendations emphasize a larger intake of lean protein sources, whole grains, and fruits to meet the adequate intake of essential fatty acids, magnesium, and zinc (19). These micronutrients are required for normal physiological functioning and the adoption of a healthy eating pattern that meets dietary recommendations is important to prevent or slow depressive symptoms and promote ideal mental and physical health (19).

Depression is associated with increased morbidity and mortality and has many economic and social consequences. Economic costs due to depression have increased the health system’s capacity to deal with the surge in mental illness (21). This can lead to stress-related disorders, further mental health damage, and increased economic costs (21).

Many micronutrients such as B vitamins, vitamin D, zinc, and magnesium are modifiable risk factors for depression (8, 10–15, 18, 20). This can explain the gaps in the literature for the mechanisms of a sufficient or insufficient balanced diet for the brains of individuals with depression (14, 17–19). Supplementation with vitamin B6 may improve elevated depressive symptoms and has been reported to increase the efficacy of antidepressants or other psychiatric medications (12, 14, 22). In previous research, a larger intake of vitamin B6 was shown to be positively associated with the onset of depression and anxiety behaviors and, additionally, can influence behavioral outcomes related to inhibition and other stress-related disorders (12, 14). However, it is important to note that diet is a modifiable risk factor that is associated with the reduction of chronic disease risks such as depression (10). Prior literature reports that magnesium supplementation has been linked to improvements in mild to moderate depression in adults and is well tolerated (10). Low magnesium intake has been correlated with an increase in depressive symptoms particularly in young adults (11).

Vitamin D has been at the center of research, along with vitamin B6 and magnesium. Studies have shown that a lack of these vitamins can be detrimental to one’s health, especially if they are undernourished and lack the consumption of these vitamins and minerals. Many individuals who have depression are reported to have insufficient levels of vitamin D, B6, and magnesium (8, 10–15, 18, 20). Therefore, examining the relationship between single micronutrient supplementation and health outcomes gives insufficient results because of the dependence on one micronutrient (19). Magnesium plays an important role in many pathways involved in the pathophysiology of depression and is found in several enzymes (11).

Treatment of depression primarily targets biological and psychological pathways (21, 23, 24). Studies have suggested that lifestyle factors such as diet quality contribute to mental disorders and play an important role in the risk of depression (2, 9, 15, 16, 21, 23). A combination of healthy dietary practices may reduce the risk of depression and provide benefits for obesity, cardiovascular diseases, and other metabolic syndromes (7). Micronutrients are heavily involved in metabolic pathways that impact the development of the functioning of the central nervous system, including brain function.

Many types of micronutrients, such as vitamins D, B6, and magnesium, help with an individual’s mental health (8, 10–14). Research has shown that vitamin D possibly controls levels of inflammatory cytokines and systemic inflammation, among others (13). Several studies found that depressed individuals had lower vitamin D levels than others, and those with the lowest levels had the greatest risk of depression depending on dosage (8, 10–14, 22–24). Studies also revealed that an increase in vitamin D and magnesium intake had a significant relationship with cognitive function (25). B vitamins play an integral role in many molecular processes that are essential for the nervous system and brain function, including many that help maintain an appropriate balance for inhibition (12, 14, 26–28).

Therefore, the primary objective of this study was to understand the aspects of dietary intake with supplementation of vitamin D, vitamin B6, and magnesium measured by the micronutrient levels measured from the NHANES dietary reports, on elevated depressive symptoms in adults aged 20 years and older living in the US. Additionally, the study aims to identify which vitamins or minerals can affect the presence of elevated depressive symptoms.

## 2 Materials and methods

### 2.1 Research questions

The research question for this study is “Does the supplementation of vitamin D, vitamin B6, and magnesium impact elevated depressive symptoms in adults aged 20 years and older living in the US?” The hypothesis of the study was to address the effects of supplementation of vitamin D, vitamin B6, and magnesium of elevated depressive symptoms among adults aged 20 years and older living in the US.

### 2.2 Study design and subject recruitment

Data from the National Health and Nutrition Examination Survey (NHANES) 2017-March 2020 survey was used to conduct this cross-sectional analysis (29). NHANES is “a series of studies to assess the health and nutritional status of adults and children in the United States” (29). NHANES uses a complex multistage stratified sampling design (29). The NHANES sample population is the noninstitutional U.S. citizens of all ages residing in all 50 states including Washington D.C. The survey examines a nationally representative sample of about 5,000 individuals per year based on interviews, physical examinations, and physical activity tests (29).

### 2.3 Eligibility criteria

#### 2.3.1 Inclusion criteria

The inclusion criteria for the study were those who completed questions related to the topic. Responses were gathered from the Dietary Supplement Use 30-Day-Total Dietary Supplements, Mental Health-Depression Screener, and Food Security sections of the dataset and were older than 19 years of age, as 20 was the minimum age respondents were required to report their marital status.

#### 2.3.2 Exclusion criteria

Before screening, individuals who reported they were pregnant and women who reported lactating were excluded to avoid bias toward depression-related pregnancy. Individuals who reported any chronic diseases were also excluded to remove additional depression-related symptoms from these conditions. Participants who did not answer the Dietary Supplement and Prescription Medicine Questionnaire (DSMQ) also were excluded from the study. It is important to note that the 2017-March 2020 dataset combines the 2017–2018 and 2019-March 2020 datasets (27).

### 2.4 Data collection

A publicly available dataset was used for a cross-sectional analysis from the NHANES 2017- March 2020 pre-pandemic dataset (30–32). Data was used from the Demographics, DSMQ, and Questionnaire Data (30–32). Questionnaire data used the nine-item depression screening instrument for the Mental Health-Depression Screening to determine the frequency of depression screening scores (30–33). The main predictor variable was the micronutrient intakes for vitamin B6 in milligrams (mg), vitamin D (D2 + D3) in micrograms (mcg), and magnesium in milligrams (mg) from the DSMQ. Intake was used because of the unreliability of serum concentrations and because it is directly modifiable and could serve as an intervention (11). Other demographic variables such as age at screening, gender, race/ethnicity, the highest level of education, marital status, and the ratio of family income to poverty were used as covariates. Household and adult food security variables were also used for analysis as potential covariates for the independent and dependent variables.

The demographic variables listed were recoded to show only individuals 20 years and older per the minimum age to report marital status. Since the PHQ-9 is used as a severity measure, the PHQ-9 score can typically range from 0 to 27, since each of the nine items can be scored from 0 (not at all) to 3 (nearly every day) (29, 33, 34). An item was also added to the end of the diagnostic portion of the PHQ-9 asking patients who checked off any problems on the questionnaire (29, 33, 34). A binary variable indicating no depression (PHQ-9 score < 10) or elevated depressive symptoms (PHQ-9 score ≥ 10) was created using a threshold score of 10 (29, 33, 34). The 10 depression screening questions were calculated into a depression screening score with values of 0 or 100. They were recoded as a binary variable as “elevated depressive symptoms (100)” or “no depression (0)” (33, 34).

### 2.5 Statistical analysis

All examinations were completed through SAS software (version 9.4; SAS Institute, Cary, NC). SAS was used to compute the values for the variables vitamin D, vitamin B6, and magnesium to reflect the median value of the responses, thus creating a binary variable for simple analysis. SAS computed the values for the variable poverty to reflect the median value of the responses. The median value for the dietary intake of vitamin D was 17.5 mcg, vitamin B6 was 2 mg, and magnesium was 50 mg, respectively. A Chi-square Test of Independence was used to examine the relationship between the supplementation of vitamins D and B6 and magnesium and the depression screening score. Additionally, a logistic regression analysis was completed to assess the nutritional status of magnesium and vitamins D and B6 against the depression screening score when adjusted for age, sex, race, and socioeconomic status (30, 31, 35). All analyses used for the stratification and weighting guidelines recommended for NHANES by the National Center for Health Statistics (29). *P* < 0.05 was used to account for statistical significance.

## 3 Results

### 3.1 Demographics

Baseline characteristics of the study population included the NHANES study participants’ demographic, dietary, and socioeconomic factors (*n* = 8,544). Among the respondents at baseline, 14.70% (*n* = 837) reported elevated depressive symptoms during the 2 weeks leading up to their physical examination, while 85.30% responded with elevated depressive symptoms. The average age was 48.41 years. Individuals who responded to the elevated depressive symptoms were married (*n* = 365; 65.14%), white, non-Hispanic (*n* = 318; 63.60%), and female (*n* = 512; 60.86%). A larger number of individuals who disclosed elevated depressive symptoms were in the “Some college or AA degree” category (*n* = 299; 34.88%), whereas “Less than 9th grade” was the lowest (*n* = 80; 5.11%). Participants who responded to having elevated depressive symptoms were categorized in the low ratio of family income to poverty (*n* = 464; 51.64%). Table 1 summarizes the demographic and socioeconomic characteristics of the study population stratified by the depression screening score, while Table 2 outlines the dietary characteristics.

**Table 1:** Characteristics of individuals’ demographic and socioeconomic status by the presence of symptoms indicated by vitamin D, B6, and magnesium dosages, NHANES (*n* = 8,544).

	Total (*n* = 8,544)	Elevated depressive symptoms	
		Yes	No
Age at screening^a^, mean (SD)	48.41 (0.52)	46.44 (0.52)	47.70 (0.66)
Gender^a^, *n* (SD), *n* (%)			
Male	4,135 (48.12)	325 (39.14)	2022 (44.52)
Female	4,409 (0.79)	512 (60.86)	2,379 (55.48)
Race/Ethnicity^a^, *n* (%), *n* (%)			
White, non-Hispanic	2,932 (2.44)	318 (63.60)	1,648 (64.76)
Black, non-Hispanic	2,291 (1.44)	216 (10.99)	1,151 (10.85)
Hispanic	1875 (1.53)	190 (15.27)	946 (15.67)
Asian, non-Hispanic	1,037 (0.91)	42 (2.51)	434 (4.68)
Other race, non-Hispanic	409 (0.37)	71 (7.63)	222 (4.04)
Marital Status, *n* (SD), *n* (%)			
Married/Living with partner	4,919 (0.93)	365 (65.14)	2,469 (75.91)
Not Married/Widowed/Divorced/Separated	1951 (0.93)	272 (34.86)	1,018 (24.09)
Education, *n* (SD), *n* (%)			
Less than 9th Grade	666 (0.38)	80 (5.11)	254 (2.77)
9-11th Grade (12th without diploma)	947 (0.36)	123 (9.56)	461 (6.70)
HS graduate/GED or equivalent	2060 (1.31)	232 (32.13)	1,052 (26.51)
Some college or AA degree	2087 (0.96)	299 (34.88)	1,535 (31.51)
College graduate or above	8,531 (2.05)	101 (18.32)	1,097 (32.51)
Ratio of family income to poverty, *n* (SD), *n* (%)			
Low poverty	3,312 (1.08)	464 (51.64)	376 (34.90)
High poverty	4,037 (1.08)	256 (48.36)	706 (65.10)
Household food security, *n* (SD), *n* (%)			
Full security: 0	4,955 (1.19)	310 (47.92)	2,567 (72.04)
Marginal security: 1–2	1,151 (0.60)	124 (14.15)	610 (11.36)
Low security: 3–5 (w/o child)/ 3–7 (w/ child)	1,146 (0.62)	163 (18.46)	599 (10.25)
Very low security: 6–10 (w/o child) 8–18 (w/child)	750 (0.49)	188 (19.47)	379 (6.35)
Adult food security, *n* (%)			
Full security: 0	4,988 (1.18)	312 (48.30)	2,588 (72.48)
Marginal security: 1–2	1,193 (0.65)	132 (14.57)	624 (11.44)
Low security: 3–5	1,023 (0.51)	140 (16.09)	535 (8.74)
Very low security: 6–10	798 (0.58)	201 (21.03)	408 (7.35)
Depression, *n* (%)			
No Symptoms of Depression	4,401 (85.30)		
Symptoms of Depression	837 (14.70)		

*N* = 837 reflects the number of individuals who responded as having elevated depressive symptoms and *N* = 4,401 reflects the number of participants who responded as not having elevated depressive symptoms. The definition of vitamin D for the study is defined as low vitamin D < 17.5 mcg whereas high vitamin D ≥ 17.5 mcg. The definition of vitamin B6 for the study is defined as low vitamin B6 < 2 mg whereas high vitamin B6 ≥ 2 mg. The definition of magnesium for the study is defined as low magnesium < 50 mg whereas high magnesium ≥ 2 mg. ^a^There were no missing data for this variable. ^b^Data were from the NHANES study.

**Table 2:** Characteristics of individuals by the presence of symptoms indicated by vitamin D, B6, and magnesium dosages, NHANES (*n* = 8,544).

	Total (*n* = 8,544)	Elevated depressive symptoms		Statistics	*p*
		Yes	No		
Vitamin D, *n* (SD), *n* (%)				*χ*^2^ = 3.6661	*p* = 0.0555
Low	1,133 (1.29)	94 (25.44)	612 (33.56)		
High	2,160 (1.29)	210 (74.56)	1,123 (66.44)		
Vitamin B6, *n* (SD), *n* (%)				*χ*^2^ = 4.4207	*p =* 0.0355
Low	749 (1.60)	56 (20.07)	417 (29.75)		
High	1810 (1.60)	162 (79.93)	946 (70.25)		
Magnesium, *n* (SD), *n* (%)				*χ*^2^ = 45.1914	*p =* 0.0227
Low	664 (2.04)	53 (23.54)	376 (34.90)		
High	1,380 (2.04)	137 (76.46)	706 (65.10)		

*N* = 837 reflects the number of individuals who responded as having elevated depressive symptoms and *N* = 4,401 reflects the number of participants who responded as not having elevated depressive symptoms. The definition of vitamin D for the study is defined as low vitamin D < 17.5 mcg whereas high vitamin D ≥ 17.5 mcg. The definition of vitamin B6 for the study is defined as low vitamin B6 < 2 mg whereas high vitamin B6 ≥ 2 mg. The definition of magnesium for the study is defined as low magnesium < 50 mg whereas high magnesium ≥ 2 mg. ^a^There were no missing data for this variable. ^b^Data were from the NHANES study.

### 3.2 Vitamin intake and depression

Chi-square analysis showed a significant association between the depression score and a few micronutrients. The analysis showed a significant association between vitamin B6 and the depression screening score (*χ*2 = 4.4207, *p* = 0.0355). Individuals (*n* = 56; 8.19%) with low vitamin B6 displayed elevated depressive symptoms. The analysis also showed a significant association between magnesium and the depression screening score (*χ*2 = 45.1914, *p* = 0.00227). Additionally, individuals (*n* = 53; 9.03%) low magnesium intake expressed having elevated depressive symptoms. Lastly, vitamin D was borderline statistically significant with the depression screening score (*χ*2 = 3.6661, *p* = 0.0555) with individuals (*n* = 94; 9.40%) reporting low vitamin D and elevated depressive symptoms.

The results of the logistic regression analysis examined the relationship between elevated depressive symptoms and the dietary intake of vitamins D, B6, and magnesium. The unadjusted model showed that low vitamin B6 correlates with an increase in the depression symptom score (OR = 0.593, 95% CI 0.355–0.991) and that low magnesium correlates with an increase in elevated depressive symptoms (Magnesium: OR = 0.574, 95% CI 0.347–0.951). The age-adjusted model showed that low vitamin D, low vitamin B6, and magnesium showed a correlation on the depression screening score (Vitamin D: OR = 0.611, 95% CI 0.382–0.980 Vitamin B6: OR = 0.503, 95% CI 0.291–0.867 Magnesium: OR = 0.458, 95% CI 0.277–0.759).

However, the fully adjusted regression model shows that low intake of vitamin B6 and magnesium correlated with an increase in the depression symptom score (Vitamin B6: OR = 0.439, 95% CI 0.260–0.738 Magnesium: OR = 0.465, 95% CI 0.303–0.714). When adjusted for all covariates, vitamin D was not statistically significant with the depression symptom score (Vitamin D: OR = 1.613, 95% CI 0.991–2.625). Table 3 overviews the significant and insignificant odds ratio and confidence intervals used to find an association between dietary intake and the depression symptom score.

**Table 3:** Odds ratios and 95% CIs against the association between vitamin D, B6, and magnesium on symptoms of depression among individuals over the age of 20, NHANES (*n* = 8,544).

	Elevated depressive symptoms					
	Unadjusted model^a^		Adjusted model^b^		Adjusted model^c^	
	OR	95% CI	OR	95% CI	OR	95% CI
High Vitamin D	1.00	(reference)	1.00	(reference)	1.00	(reference)
Low Vitamin D	0.676	(0.442–1.032)	0.611	(0.382–0.980)	0.620	(0.381–1.009)
High Vitamin B6	1.00	(reference)	1.00	(reference)	1.00	(reference)
Low Vitamin B6	0.593	(0.355–0.991)	0.503	(0.291–0.867)	0.439	(0.260–0.738)
High Magnesium	1.00	(reference)	1.00	(reference)	1.00	(reference)
Low Magnesium	0.574	(0.347–0.951)	0.458	(0.277–0.759)	0.465	(0.303–0.714)

^a^Not adjusted for any covariates; ^b^Age-adjusted model; ^c^Adjusted for the following covariates: age at screening, gender, race/ethnicity, marital status, education, the ratio of family income to poverty, household food security, and adult food security. The definition of vitamin D for the study is defined as low vitamin D < 17.5 mcg whereas high vitamin D ≥ 17.5 mcg. The definition of vitamin B6 for the study is defined as low vitamin B6 < 2 mg whereas high vitamin B6 ≥ 2 mg. The definition of magnesium for the study is defined as low magnesium <50 mg whereas high magnesium ≥2 mg.

The results of the fully adjusted model showed no association between the symptoms of depression and a lower intake of vitamin D when adjusted for covariates (Vitamin D: OR = 0.981, 95% CI 0.476–2.020). Moreover, when adjusted for all covariates, low magnesium showed a relationship with symptoms of depression (Magnesium: OR = 0.846 95% CI 0.389–1.839). When adjusted for all covariates, low intake of vitamin B6 showed a correlation for symptoms of depression (Vitamin B6: OR = 0.408 95% CI 0.169–0.986). Only vitamin B6 showed a relationship in the age-adjusted model when vitamin D and magnesium were included as covariates (Vitamin B6: OR = 0.439 95% CI 0.200–0.963). Odds ratios for unadjusted, age-adjusted, and fully adjusted models for the combination of vitamins D, B6, and magnesium on the elevated depressive symptoms screening score are shown in Table 4.

**Table 4:** Odds ratios and 95% CIs against the association of vitamin D, vitamin B6, and magnesium combined on symptoms of depression among individuals over the age of 20, NHANES study (*n* = 8,544).

		Elevated depressive symptoms					
		Unadjusted model^a^		Age-adjusted model^b^		Age-adjusted model^c^	
	*n*	OR	95% CI	OR	95% CI	OR	95% CI
High Vitamin D	210	1.00	(reference)	1.00	(reference)	1.00	(reference)
High Vitamin B6	162	1.00		1.00		1.00	
High Magnesium	137	1.00		1.00		1.00	
Low Vitamin D	94	0.866	(0.484–1.551)	0.787	(0.426–1.454)	0.981	(0.476–2.020)
Low Vitamin B6	56	0.471	(0.211–1.054)	0.439	(0.200–0.963)	0.408	(0.169–0.986)
Low Magnesium	53	0.906	(0.438–1.873)	0.801	(0.395–1.622)	0.846	(0.389–1.839)

^a.^Not adjusted for any covariates; ^b.^Age-adjusted model; ^c.^Adjusted for the following covariates: age at screening, gender, race/ethnicity, marital status, education, the ratio of family income to poverty, household food security, and adult food security. *N*=Number of cases for each vitamin that reported elevated depressive symptoms. The definition of vitamin D for the study is defined as low vitamin D < 17.5 mcg whereas high vitamin D ≥ 17.5 mcg. The definition of vitamin B6 for the study is defined as low vitamin B6 < 2 mg whereas high vitamin B6 ≥ 2 mg. The definition of magnesium for the study is defined as low magnesium <50 mg whereas high magnesium ≥2 mg.

## 4 Discussion

Data from NHANES was used to evaluate whether dietary intake with vitamin D, vitamin B6, and magnesium supplementation was associated with elevated depressive symptoms in adults aged 20 years and older living in the US. The results of this cross-sectional study show a significant association between depression and vitamin B6 intake. The results of the analysis also showed that there may be an association between magnesium intake and depression. The analysis findings showed that there could be significant differences between vitamin D, vitamin B6, and magnesium related to elevated depressive symptoms when adjusting for covariates. These findings aligned with the primary objectives, where the result appears to identify which supplements may have the ability to affect elevated depressive symptoms. Low intake of vitamin B6 and magnesium may affect elevated depressive symptoms when adjusted for covariates. Additionally, when analyzing the effects of vitamin D, vitamin B6, and magnesium intake, the results showed vitamin B6 could be significant for reducing elevated depressive symptoms. However, the analysis demonstrates that vitamin B6 and magnesium intake on elevated depressive symptoms is important when considering age and other sociodemographic factors.

From current knowledge, this present study is the first to explore the specific dietary composition of vitamins and minerals and their association with elevated depressive symptoms in adults. As it is known, the common treatments for depression include antidepressant medication and psychotherapy (7, 9, 16–18, 25, 26). These results may show possible alternative methods to preventing depression and other mental health disorders through natural over-the-counter remedies rather than prescribed antidepressants. However, meeting adequate dietary intake of micronutrients for reducing elevated depressive symptoms and other mental health-related illnesses remains unclear.

Possible reasons for the findings of this study can be attributable to multiple reasons. According to prior research, the term lifestyle medicine which is defined as “the differences in lifestyle habits, such as physical activity, diet, substance use, and sleep, to improve mental health symptoms” can be a possible explanation for the findings of this study (36). Young et al. (36) researched the effects of an app-based program that can promote dietary change, and the results showed that participants who changed their diet were more likely to set goals toward changing their eating habits. Using an app such as the one used in this study can help future participants better adhere to their lifestyle changes that can help improve their elevated depressive symptoms.

Young et al. (36) tested the feasibility of the My Food and Mood mobile app to support dietary change in individuals with depression. This study reported a significant increase in the scores from baseline to halfway through the program, in which the program showed that it promoted better diet quality (36, 37). Nevertheless, the Young et al. study has limitations (36). The main limitation is that it can be improved further to increase engagement, retention, and dietary improvement (36, 37).

The positive association between low dietary intake and elevated depressive symptoms may be explained by several factors that may play a role in the psychosocial factors connected with poor nutrition (10, 11, 25–27, 37). Proposed nutritional deficiencies and treatments for persons with major depression, bipolar disorder, schizophrenia, and obsessive-compulsive disorder have been reported. The findings of previous studies support the results of this study by showing that there exists a relationship between vitamin deficiencies and depressive symptoms. For example, vitamin D has been linked to seasonal affective disorder (SAD), schizophrenia, and depression, in which the amount of light therapy was examined for mood changes. Partonen et al. (28) reported that about 1 hour of light therapy significantly decreases symptoms of depression in patients with SAD compared to those without light therapy. Additionally, a dose–response association between vitamin D levels and depression at their baseline measurement was outlined by Ronaldson et al. (27). This showed an increased risk of depression among participants with inadequate levels of vitamin D and adequate levels at baseline and follow-up (27). From this current study, it is important to recognize the impact of vitamin D on depression depends on the dose taken to find differences in mood.

As previous literature has stated, lifestyle factors such as smoking, sedentary behavior, and alcohol consumption are increasing and can lead to the development of mental health disorders (16–18). The finding that vitamin B6 supplementation reduced depression is consistent with the findings in many studies (12, 14). Looking at the biological mechanisms, B6 is among other B vitamins essential for a normal central nervous system and brain function and is shown to control moods through several pathways. This inference can be attributable to how vitamin deficiencies could potentially mimic or falsely elevate depressive symptoms (17, 18, 22, 23). B vitamins also play a role in the proper functioning of the nervous tissue (12, 14). Noah et al. (14) discovered that magnesium, with or without additional supplementation of B6, improved mood and anxiety in healthy adults, which aligns with the results of this research. The findings of the previous studies support other studies’ findings in that vitamin B6 and magnesium can be potential treatments for reducing symptoms of depression (14).

### 4.1 Strengths and limitations

Although the analysis of this study showed that there is the possibility of a difference in low intake of micronutrients against elevated depressive symptoms, there are several strengths and limitations to this study. One limitation is the major differences in individual sample sizes between the participants who reported data for each micronutrient. For example, the sample size for magnesium had significantly fewer responses compared to vitamin D, making it difficult to draw valid conclusions from the data. This limitation shows that the results cannot be generalizable for the population, as many responses were missing for each predictor variable and can be attributed to lower statistical power and reduced effect size in this study. However, the initial sample size used for this analysis was a strength in evaluating the effectiveness of sociodemographic variables in the regression analysis.

Furthermore, there is a possibility of survey bias for responses of the predictor variables, in which many respondents were not candid on their questionnaire. Even so, NHANES used a Food Frequency Questionnaire (FFQ) to assess all the vitamins and minerals. FFQs are not always valid to determine if the recommended daily intake of essential vitamins and minerals is met.

Another limitation of the study was that there is no standard measurement for the amount of vitamin D, vitamin B6, and magnesium intake. Additionally, the dataset did not indicate how each vitamin and mineral intake was calculated based on individual dietary or supplement intakes. In contrast, this study did not have evidence that showed changes in symptoms from the use of vitamin D. Future studies are required to clarify the association of vitamin D and depression, as some of the literature shows there is an association while others do not (13, 15, 26, 27). Case–control or prospective cohort studies may be valuable for understanding the precise effect vitamins D, B6 and magnesium have on the prevalence of depression screening scores.

## 5 Conclusion

The nature of one’s diet and its repercussions on health are characterized in mental health as a public health problem. Numerous studies provide reassurance for the direct relationship between nutrition, stress susceptibility, mental health, and mental function. The reviewed literature expressed the importance of vitamins, and minerals included in the prevention and treatment of symptoms of depression (8, 10–15, 18, 20). Research shows that many individuals who reported elevated depressive symptoms have a nutrient deficiency (8, 10–15, 18, 20, 24–27). However, the relationship between diet, obesity, stress, and stress-related psychiatric disorders is complex and cannot be inferred from this cross-sectional study (12, 18, 23–25). However, prior research has exhibited the advantages of eating nutrient-rich foods on an individual’s overall health (11, 12, 14, 15, 26).

The analysis supports this study’s hypothesis that there may be a relationship between varying intakes of vitamins and minerals on elevated depressive symptoms. This association may be evident for lower vitamin B6 and magnesium intakes when adjusted for age and sociodemographic covariates. Of the two vitamins, vitamin B6 and magnesium, vitamin B6 revealed the most affecting factor. These analysis findings indicate the need for further research on the impact of diet on mental health. Future prospective cohort studies exploring these associations, focusing on daily dietary intake, are needed to validate the direction of causation further and understand the underlying mechanisms.

## Data availability statement

Publicly available datasets were analyzed in this study. This data can be found at: https://wwwn.cdc.gov/nchs/nhanes/search/datapage.aspx?Component=Questionnaire&Cycle=2017-2020.

## Ethics statement

The studies involving humans were approved by Grand Valley State University institutional review board. The studies were conducted in accordance with the local legislation and institutional requirements. Written informed consent for participation was not required from the participants or the participants’ legal guardians/next of kin in accordance with the national legislation and institutional requirements.

## Author contributions

RR: Conceptualization, Data curation, Formal analysis, Investigation, Methodology, Resources, Writing – original draft, Writing – review & editing, Project administration, Visualization. JV: Methodology, Resources, Supervision, Writing – review & editing, Project administration, Visualization. KB: Supervision, Writing – review & editing, Project administration, Resources. NA: Resources, Supervision, Writing – review & editing, Project administration, Visualization.
